# Author Correction: Thyroid hormone induces progression and invasiveness of squamous cell carcinomas by promoting a ZEB-1/E-cadherin switch

**DOI:** 10.1038/s41467-019-13904-w

**Published:** 2020-01-08

**Authors:** Caterina Miro, Emery Di Cicco, Raffaele Ambrosio, Giuseppina Mancino, Daniela Di Girolamo, Annunziata Gaetana Cicatiello, Serena Sagliocchi, Annarita Nappi, Maria Angela De Stefano, Cristina Luongo, Dario Antonini, Feliciano Visconte, Silvia Varricchio, Gennaro Ilardi, Luigi Del Vecchio, Stefania Staibano, Anita Boelen, Cedric Blanpain, Caterina Missero, Domenico Salvatore, Monica Dentice

**Affiliations:** 1 Department of Clinical Medicine and Surgery, University of Naples “Federico II”, Naples, Italy; 2 IRCCS SDN, Naples, Italy; 3 Department of Biology, University of Naples “Federico II”, Naples, Italy; 4 CEINGE–Biotecnologie Avanzate Scarl, Naples, Italy; 5 Department of Advanced Biomedical Sciences, University of Naples “Federico II”, Naples, Italy; 6 Endocrine Laboratory, Department of Clinical Chemistry, Amsterdam University Medical Center, location AMC, Amsterdam, The Netherlands; 7 IRIBHM, Université Libre de Bruxelles (ULB), Brussels, Belgium; 8 Department of Public Health, University of Naples “Federico II”, Naples, Italy

**Keywords:** Cancer, Cell biology, Molecular biology, Endocrinology

Correction to: *Nature Communications* 10.1038/s41467-019-13140-2, published online 27 November 2019.

The original version of this Article contained an error in the author affiliations. Silvia Varricchio, Gennaro Ilardi and Stefania Staibanow were incorrectly associated with ‘Department of Public Health, University of Naples "Federico II", Naples, Italy’ instead of the correct ‘Department of Advanced Biomedical Sciences, University of Naples “Federico II”, Naples, Italy.’ This has now been corrected in both the PDF and HTML versions of the Article.

