# Supplementary material for: Thyroid hormone induces progression and invasiveness of squamous cell carcinomas by promoting a ZEB-1/E-cadherin switch
Source: Nat Commun. 2019 Nov 27;10:5410. doi: 10.1038/s41467-019-13140-2 (PMC6881453; doi:10.1038/s41467-019-13140-2)
Supplement: Supplementary file 1 — Supplementary Information [file 41467_2019_13140_MOESM1_ESM.pdf]

Supplementary Information

Miro et al.

Thyroid Hormone Induces Progression and Invasiveness of Squamous Cell Carcinomas by Promoting a ZEB-1/E-cadherin Switch

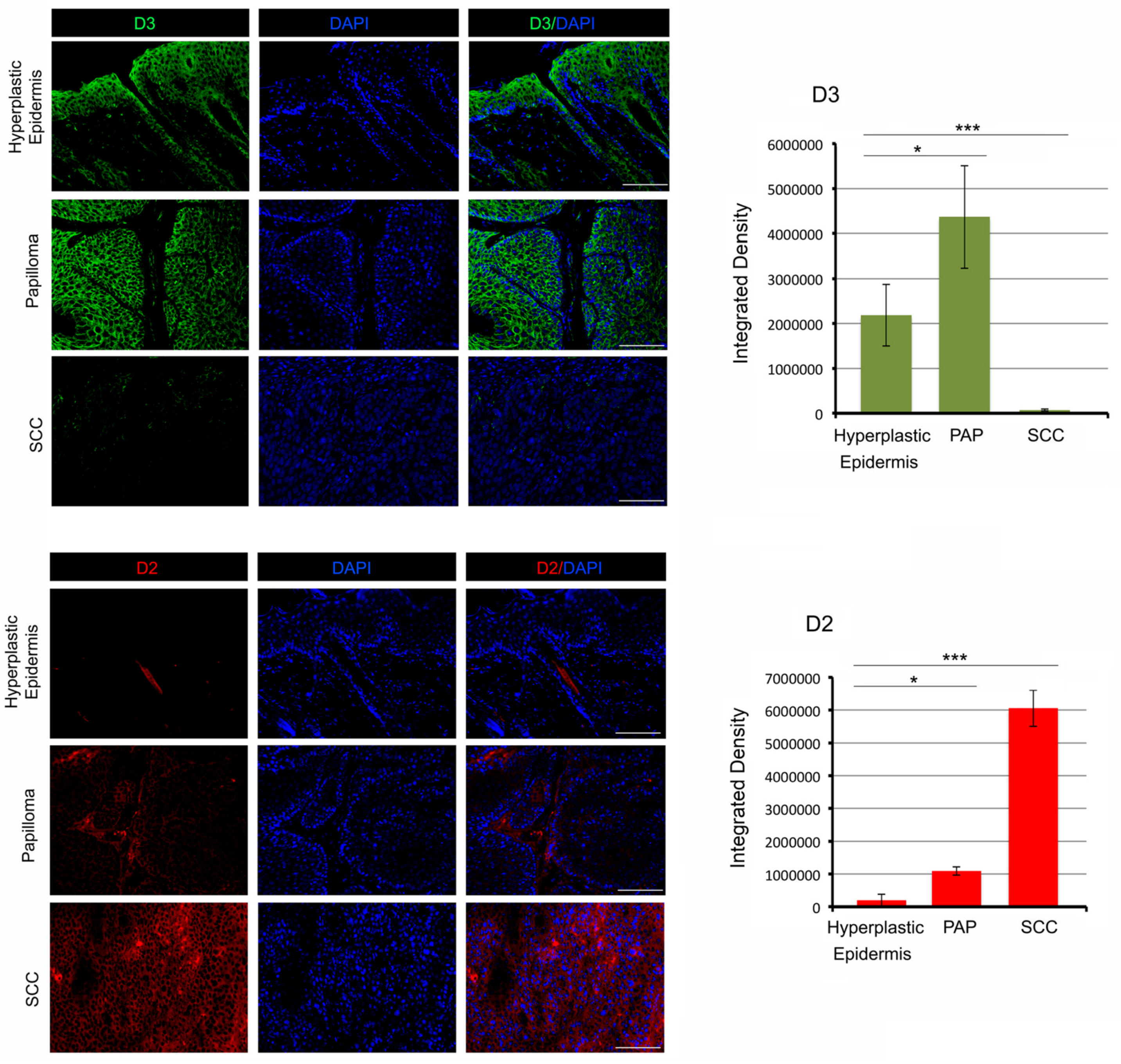

Supplementary Figure 1. *D2 and D3 are dynamically expressed in different stages of SCC tumor progression.* Immunofluorescent (IF) analysis of D2 and D3 expression in chemically induced SCC at 12, 15 and 30 weeks after DMBA treatment. Scale bars represented 100 μm.

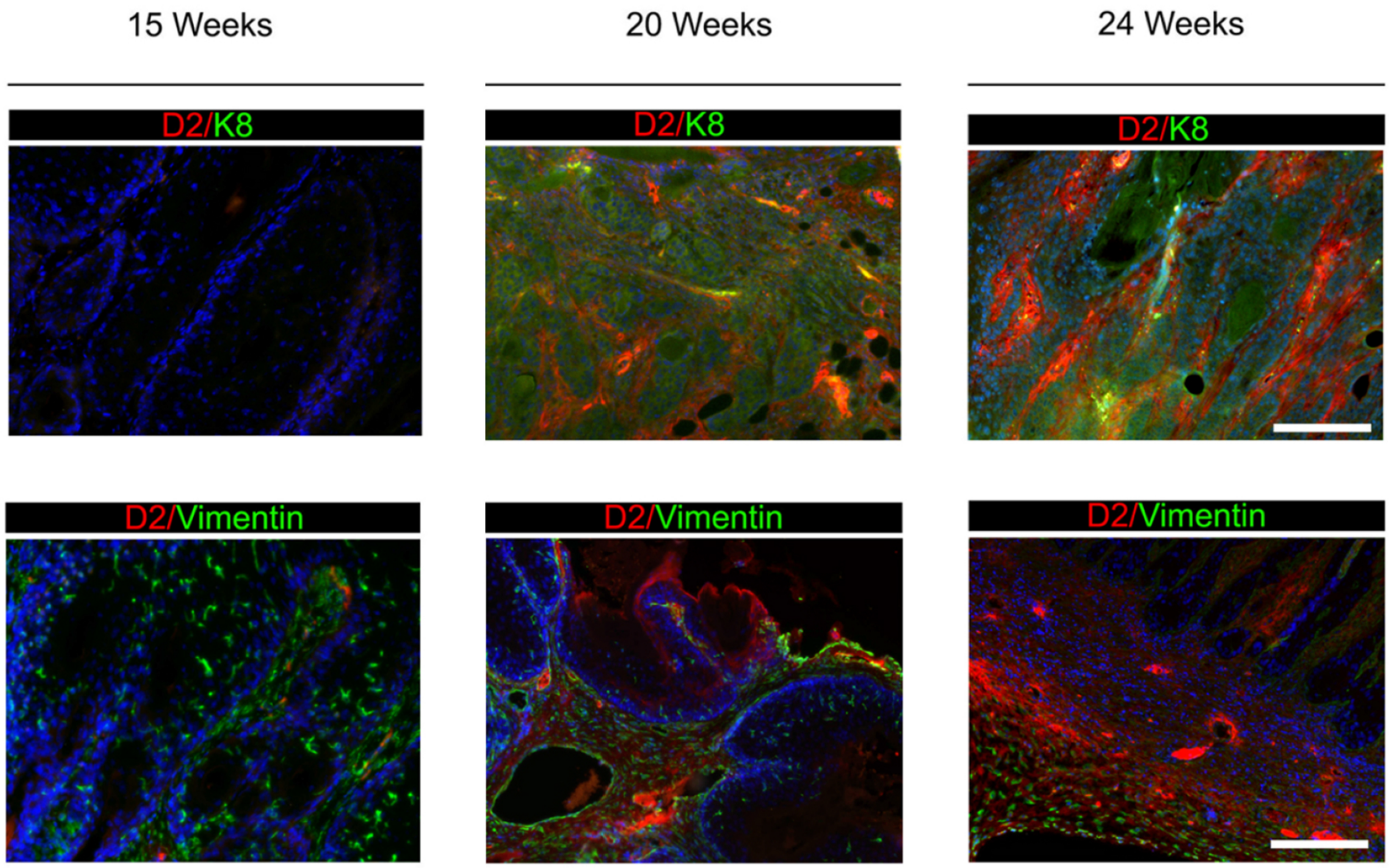

Supplementary Figure 2. *D2 expression correlates with markers SCC*. Co-Immunofluorescent analysis of D2/K8 and D2/Vimentin expression in chemically induced SCC at 15, 20 and 24 weeks after DMBA treatment. D2 co-localizes with K8 and only partially co-localizes with vimentin. Scale bars represented 200 μm.

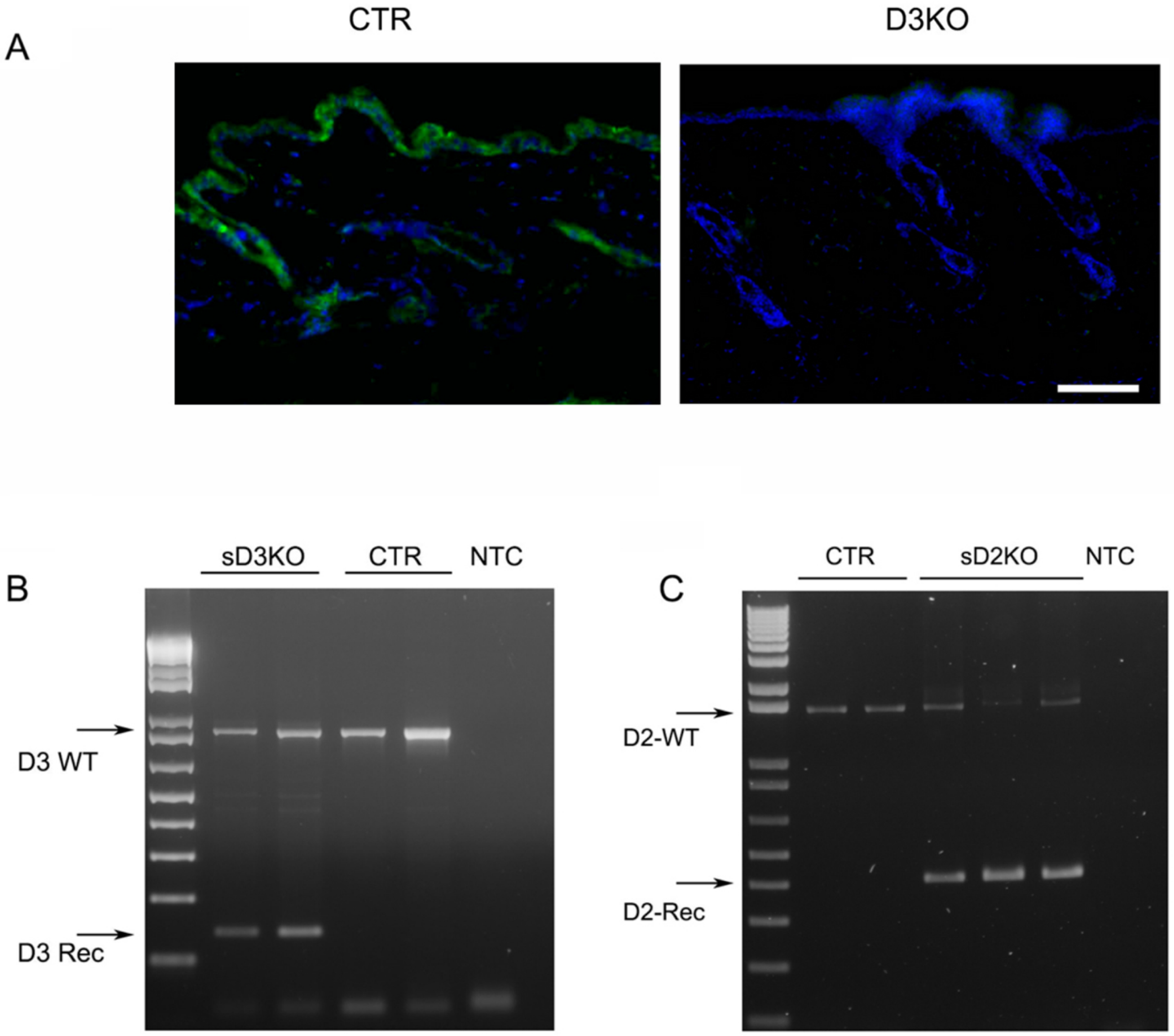

Supplementary Figure 3. *D3- and D2-depletion in the epidermal compartment.* **(A)** D3 depletion in sD3KO mice was confirmed by immunofluorescent analysis of dorsal skin of sD3KO and control mice. Scale bars represented 100 μm**. (B)** D3 depletion in sD3KO mice was confirmed by PCR analysis of the same dorsal skin as in A. **(C)** D2 depletion in sD2KO mice was confirmed by PCR analysis of the dorsal skin of sD2KO and control mice.

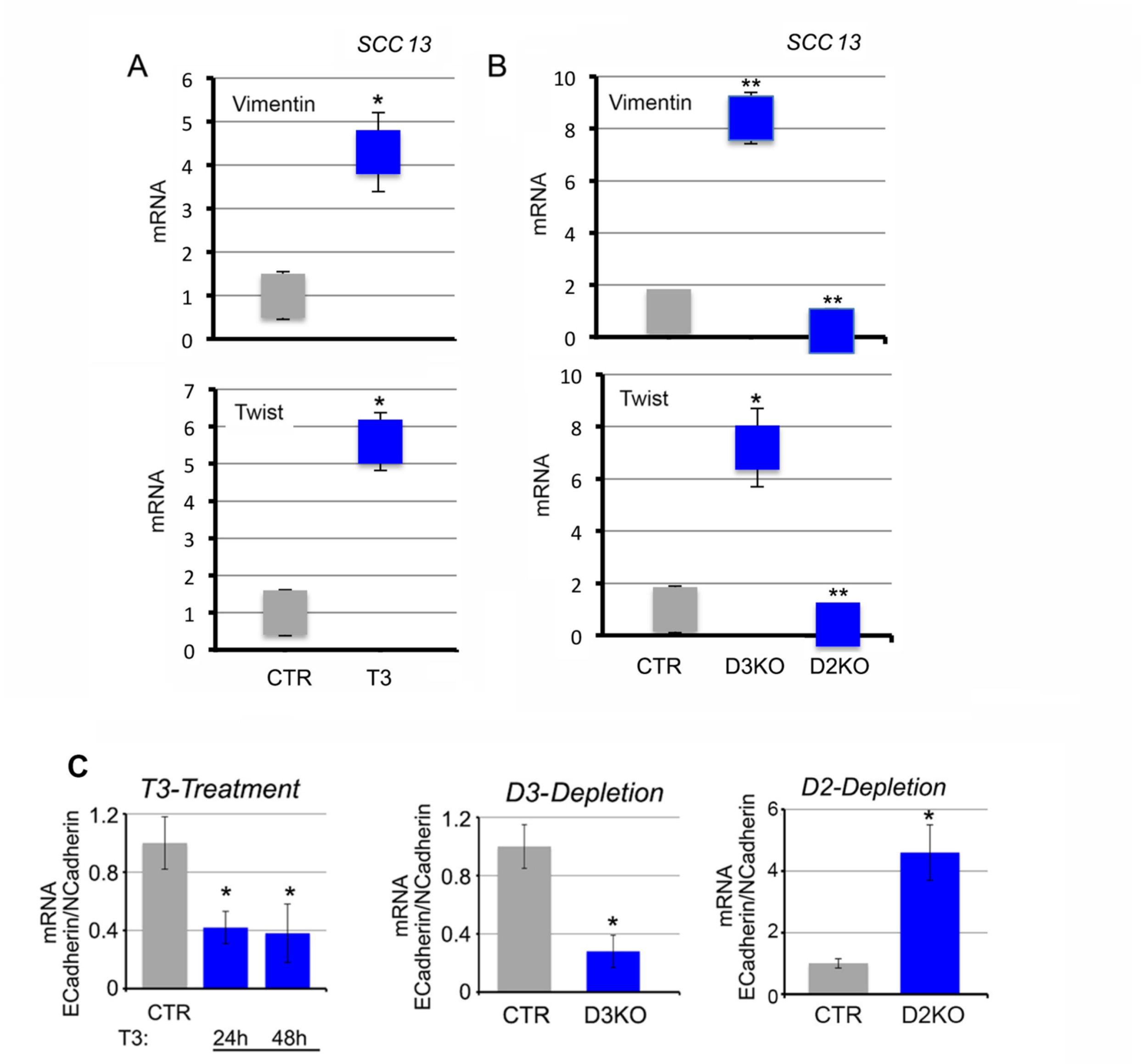

Supplementary Figure 4. *Markers of EMT are regulated by T3 and deiodinases silencing in SCC 13 cells.* **(A)** Vimentin and Twist mRNA expression in SCC cells treated with 30 nM T3 for 48h. **(B)** Vimentin and Twist mRNA in D2KO and D3KO cells compared to the control, CRISP-CTR SCC cells. **(C)** E-Cadherin/N- Cadherin ratio was evaluated by real time PCR in T3-treated and in D3KO and D2KO cells.

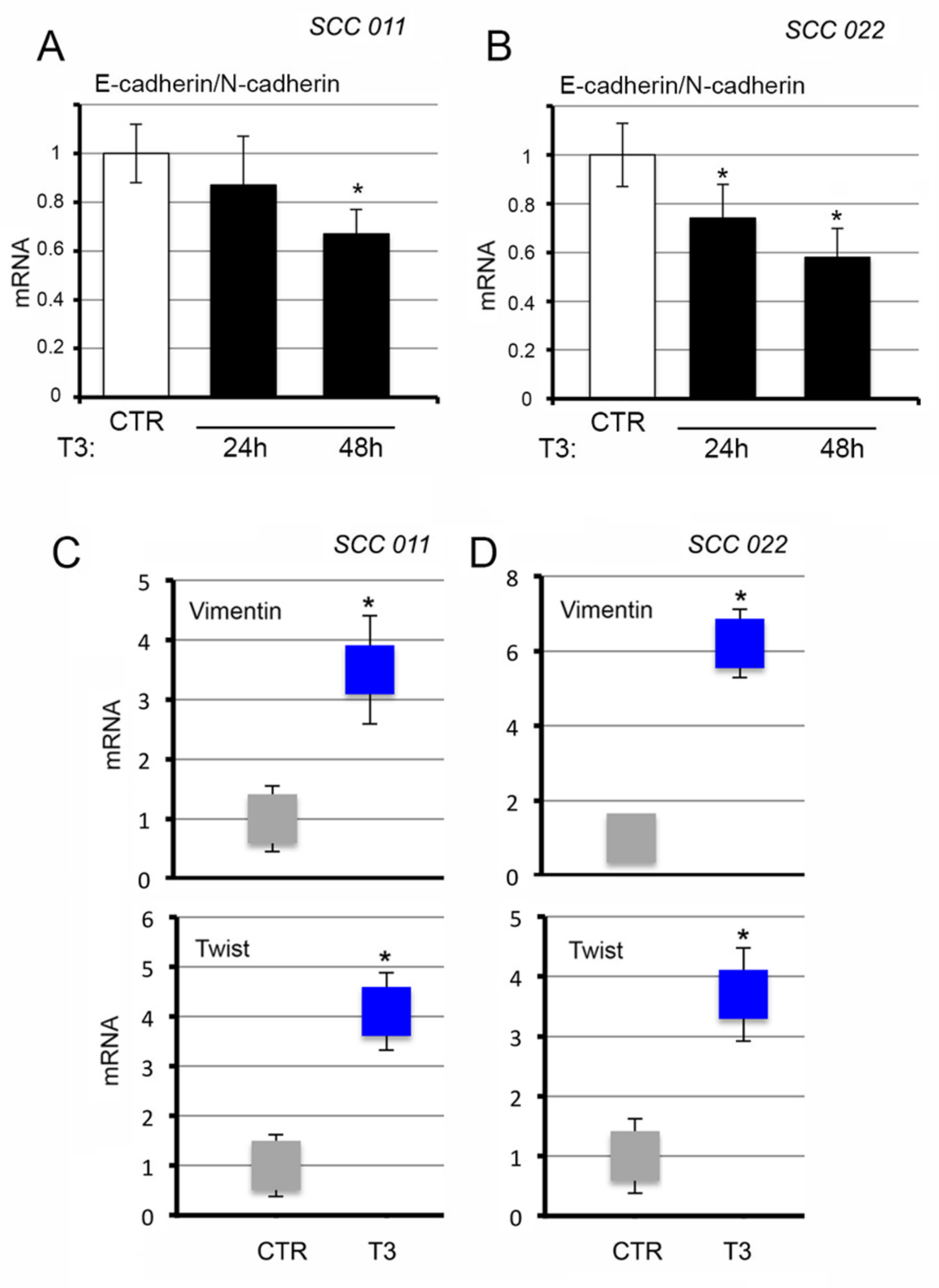

Supplementary Figure 5. *Markers of EMT are regulated by T3 and deiodinases silencing in SCC 011 and 022 cells.* **(A)** E-cadherin and N-cadherin mRNA expression and their ratio were measured by real time PCR in SCC cells treated with 30 nM T3 for 24h and 48h. **(B)** Vimentin and Twist mRNA levels were evaluated by real time PCR in T3-treated SCC 011 and SCC 022 cells.

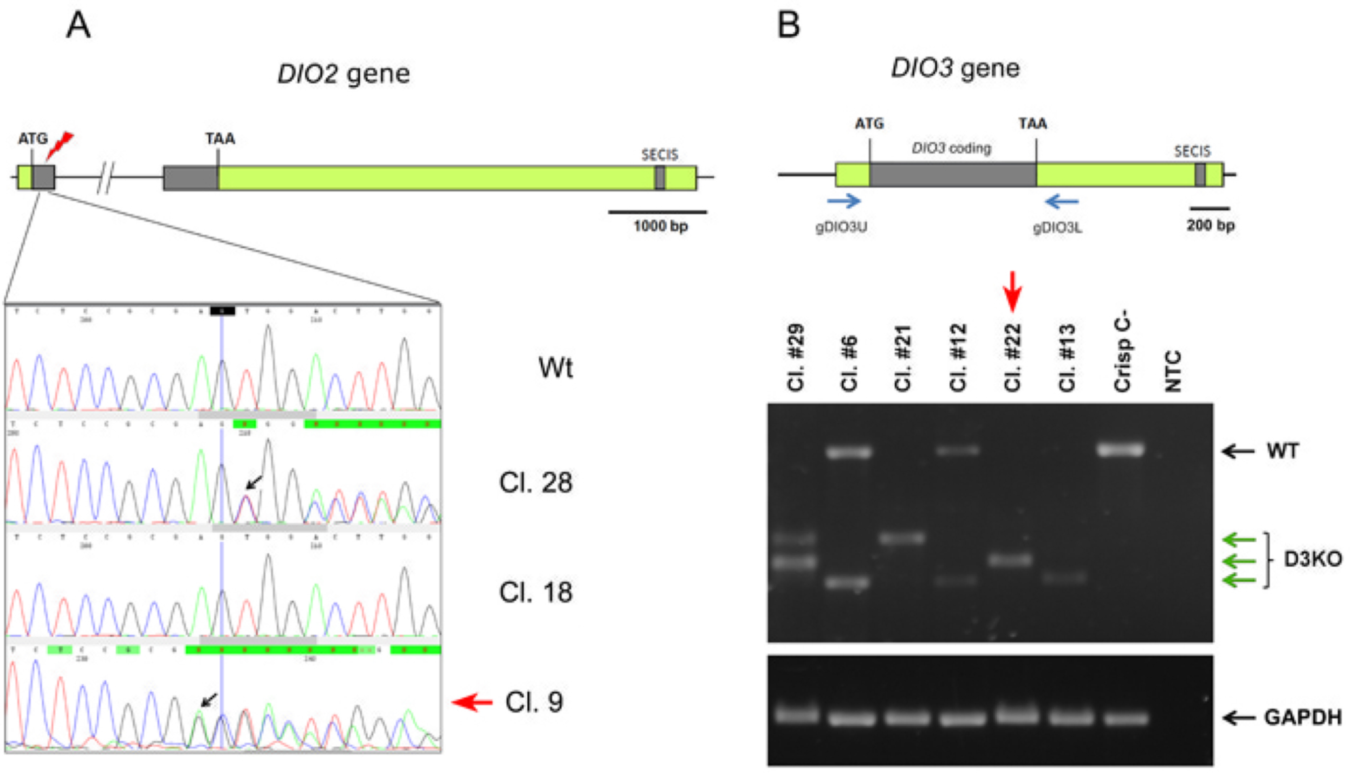

Supplementary Figure 6. *Mutagenesis of Dio2 and Dio3 gene in SCC cells.* **(A)** Schematic representation of the *DIO2* locus. Mutagenesis of *DIO2* locus was assessed by genomic DNA sequencing of exon 1. **(B)** Schematic representation of the *DIO3* locus. Mutagenesis of DIO3 locus was assessed by PCR analysis. The green arrows indicate the mutated DIO3 products.

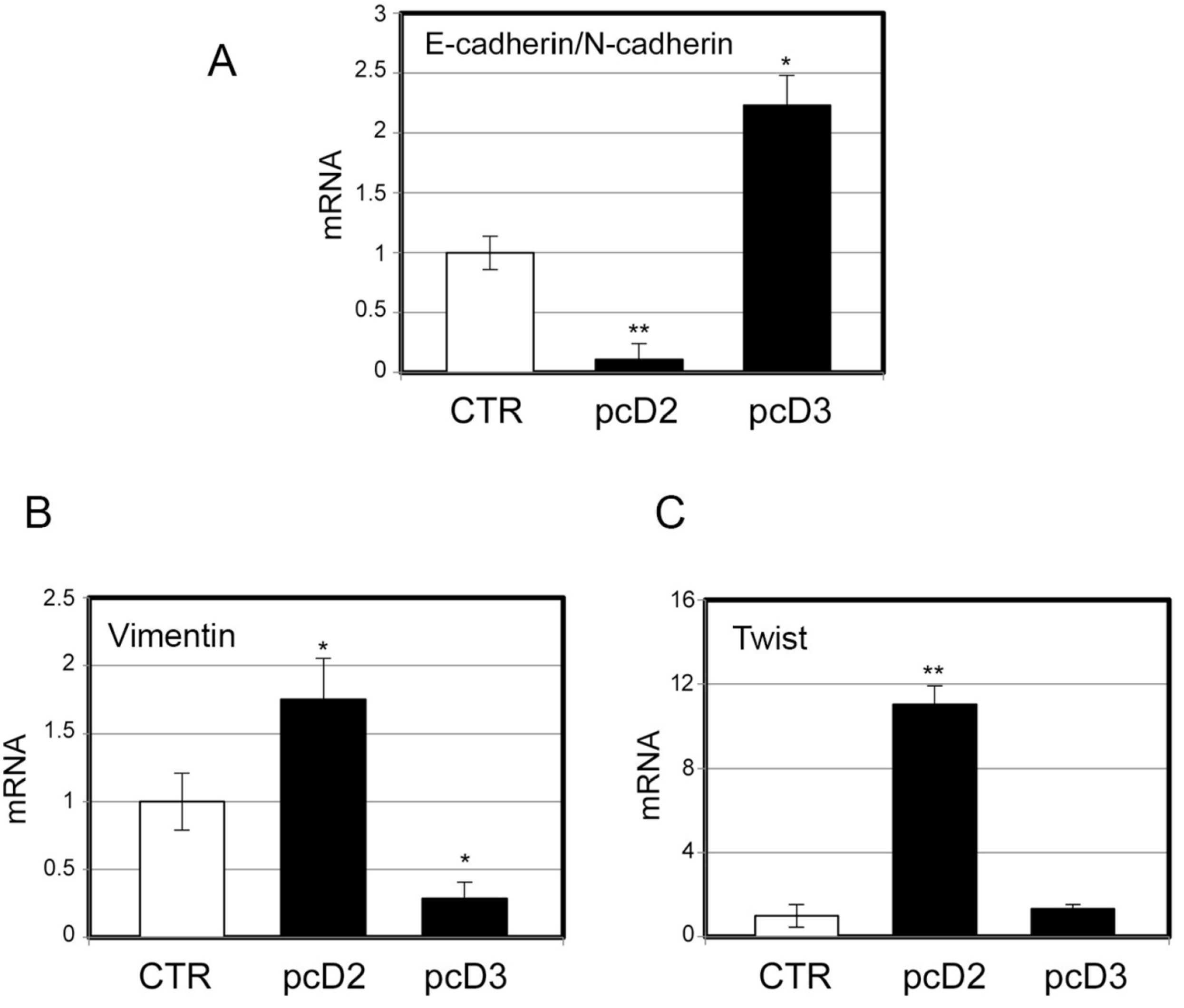

Supplementary Figure 7. *Deiodinases overexpression regulates expression of markers of EMT.* **(A)** E-cadherin and N-cadherin mRNA expression were measured by real time PCR in SCC cells transfected with D2- (pcD2) and D3-overexpression plasmid (pcD3). **(B-C)** Vimentin and Twist mRNA levels were evaluated by real time PCR in same cells as in A.

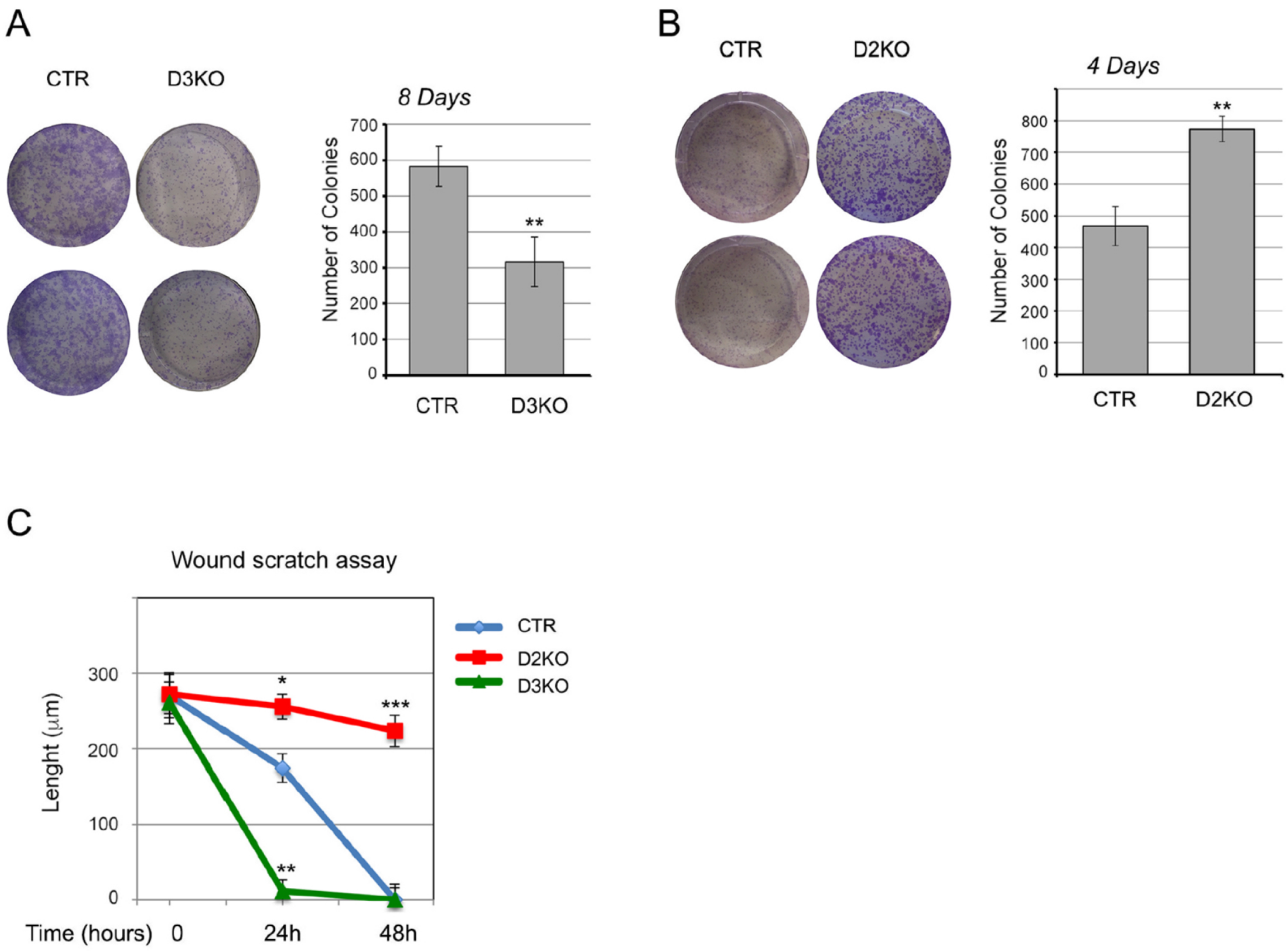

Supplementary Figure 8. *D3 and D2 Depletion inversely regulate migration of SCC cells.* **(A, B)** Clonogenic assay of CTR and D3KO (A) and CTR and D2KO (B) cells; representative images of petri dishes are shown. (**C)** Wound scratch assay was performed in CTR, D2KO and D3KO clones. Cell migration was measured as described. *p < 0.05, **p < 0.01, and ***p < 0.001.

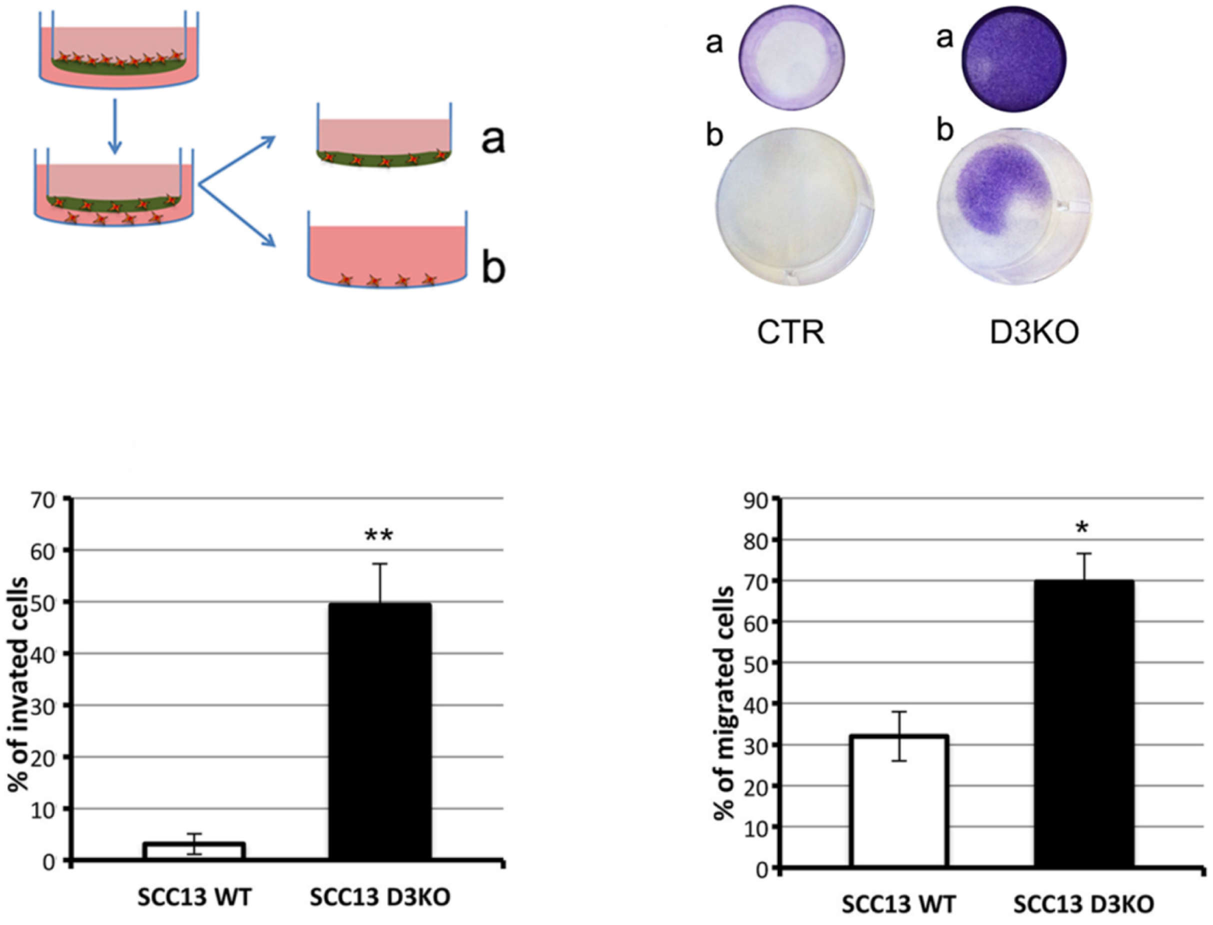

Supplementary Figure 9. *D3 Depletion increases invasiveness of SCC cells.* Invasion assay was performed on SCC CTR and D3KO cells. Cells in b represent cells invaded on the receiver plate. The percentage of the cells that migrated and invaded are represented by histograms.

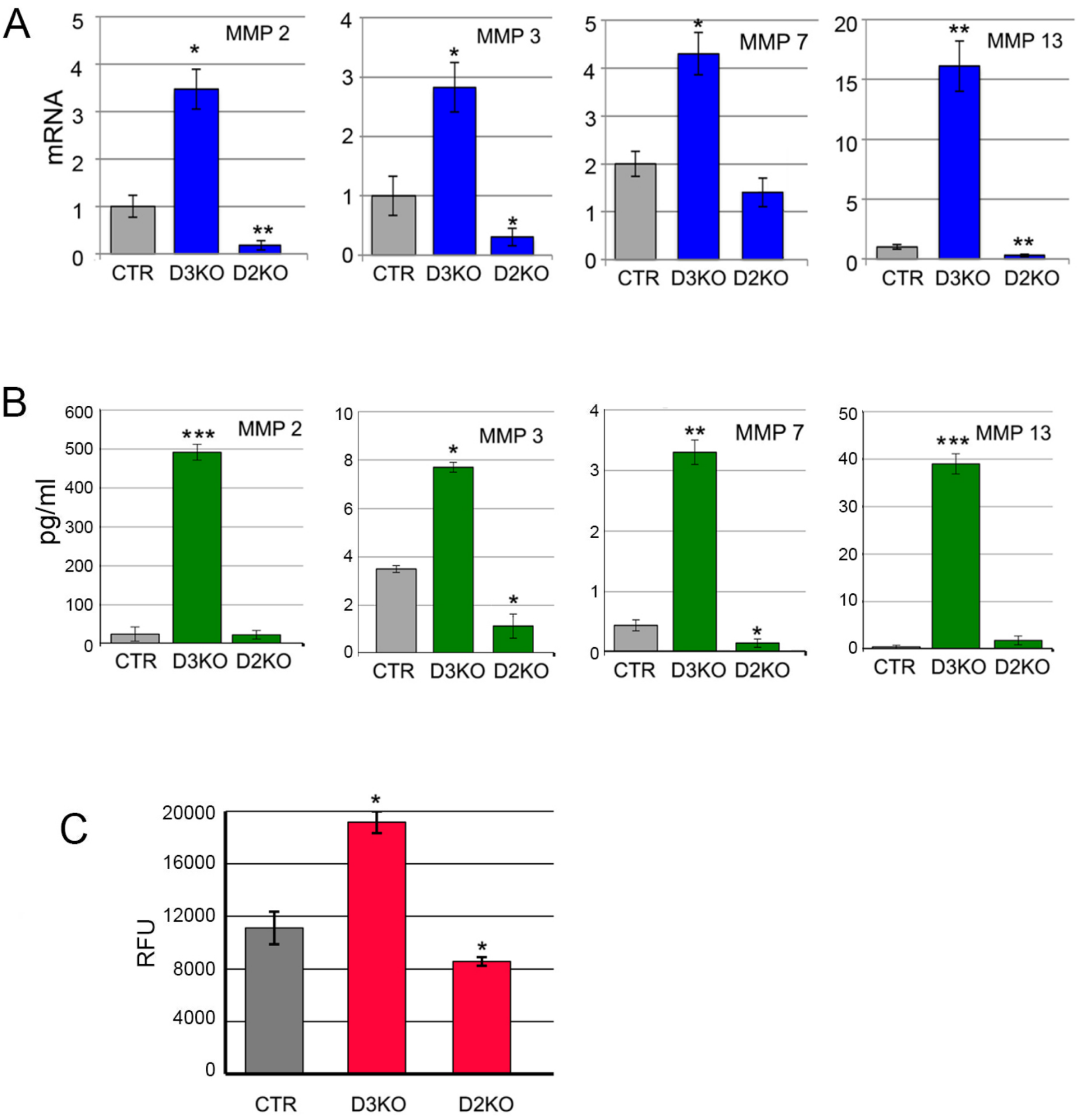

Supplementary Figure 10. *D3 Depletion increases the activity of metalloproteases.* (A) Metalloproteases 2, 3, 7 and 13 mRNA expression was analyzed by real time PCR in D3KO, D2KO and CTR cells. (B) Rate of secretion of Metalloproteases was measured in the culture medium. (C) Enzymatic activity of Metalloproteases was measured by Elisa.

A

Up-regulated genes

Gene	Fold Change	p-Value
MAP1B	8,0556	0,000033
ZEB1	7,3871	0,00023
BMP2	6,0002	0,000171
CDH2	5,0455	0,000063
TFPI2	4,4537	0,000031
VCAN	4,1267	0,006012
SPP1	3,5431	0,022297
TGFB2	2,8879	0,000249
ZEB2	2,7606	0,022277
VIM	2,4368	0,003281
COL1A2	2,2191	0,017992
GNG11	2,0209	0,000001
PLEK2	2,0209	0,000007
JAG1	1,9656	0,005
SERPINE1	1,8025	0,000001
STEAP1	1,7532	0,020452
SNAI2	1,6301	0,000481
TMEFF1	1,5966	0,005836
FZD7	1,5746	0,004339
ITGA5	1,5692	0,00003
DESI1	1,4794	0,000065
CTNNB1	1,366	0,011436

Down-regulated genes

Gene	Fold Change	p-Value
KRT14	0,3345	0,000023
DSP	0,3856	0,000137
KRT19	0,4005	0,000001
AHNAK	0,4863	0,0005
BMP1	0,4948	0,000001
VPS13A	0,5471	0,000083
CDH1	0,5586	0,001817
DSC2	0,5844	0,000033
ERBB3	0,5967	0,000077
TCF3	0,6092	0,000515
ITGAV	0,6736	0,000013
PTK2	0,6807	0,000002
TGFB1	0,6854	0,002131

B

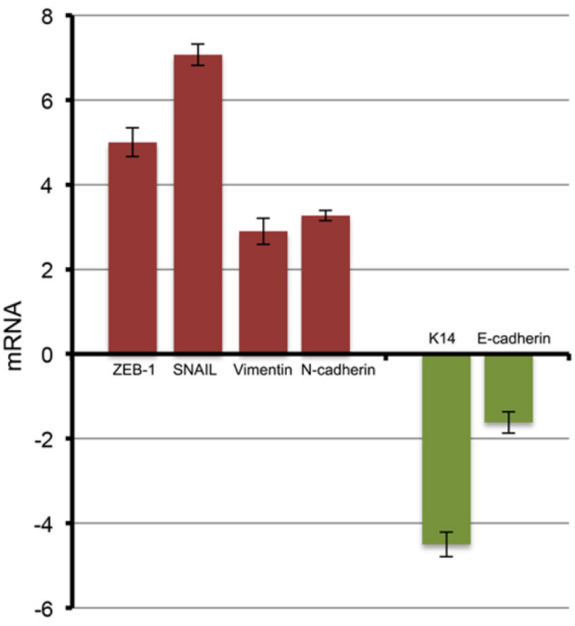

Supplementary Figure 11. *D3 Depletion increases the expression of EMT genes.* **(A)** Expression levels of up- and down-regulated EMT markers in the EMT RT2 ProfilerTM PCR Array. **(B)** T3-target genes in the EMT RT2 ProfilerTM PCR Array were validated by using Real Time PCR analysys in CTR and D3KO cells.

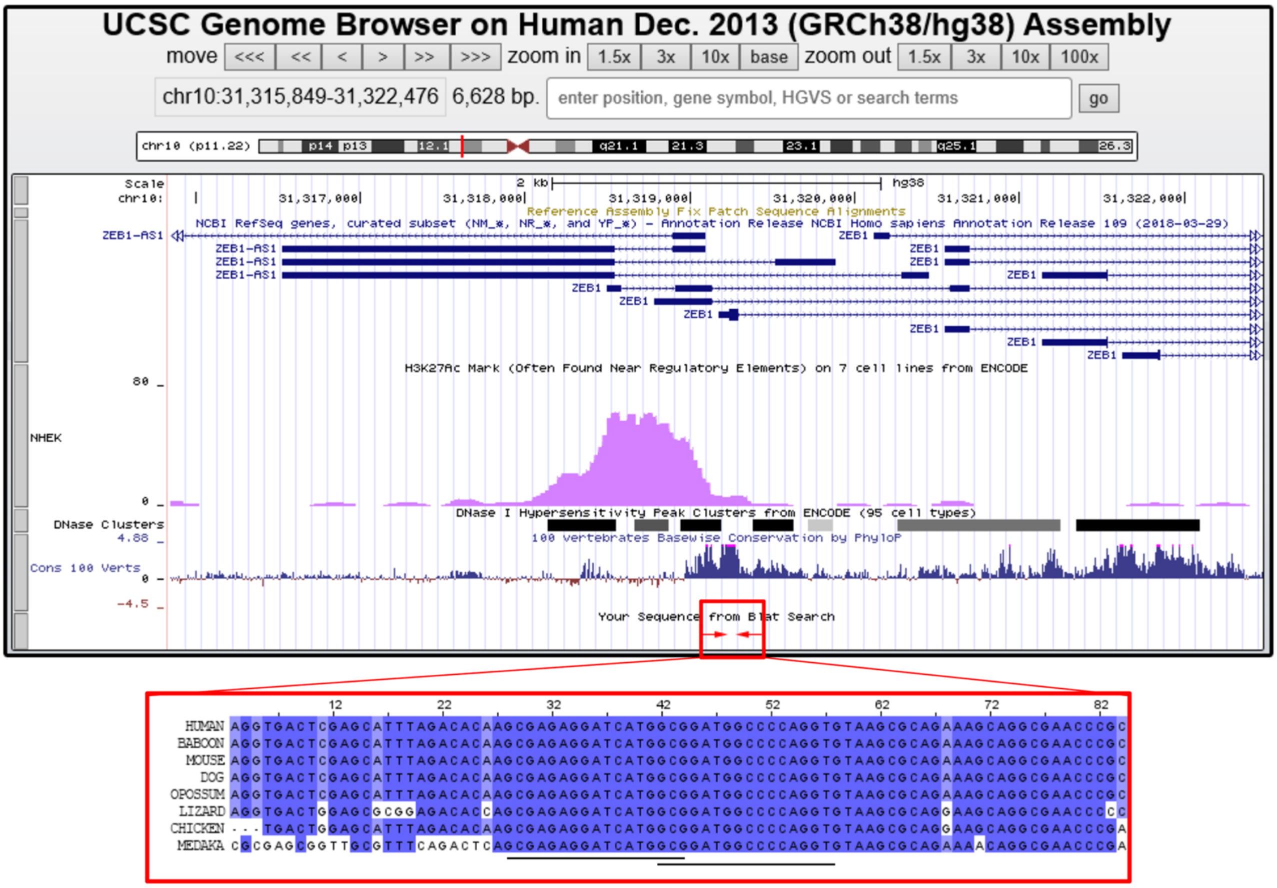

T3R_01 SNNTRAGGTCACGSNN

Supplementary Figure 12. *The 5'-Flanking region of ZEB-1 gene contains multiple T3-binding sites.* Schematic representation of human ZEB-1 5' flanking region. (UP) RefSeq genes, layered H3K27Ac in NEK cells and vertebrate conservation from the UCSC Genome Browser on Human Dec. 2013 Assembly (GRCh38/hg38). (DOWN) putative THR binding sites (V$T3R_01) from TFBind Software (strand +: red; strand - : bleu) and the validated ChIP Region (red arrows).

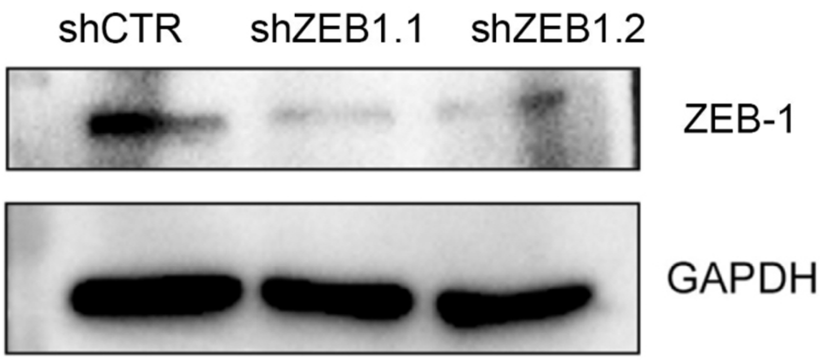

Supplementary Figure 13. *Effective ZEB-1 silencing.* Expression of endogenous ZEB-1 was measured by western blot analysis in SCC cells transfected with a scramble sh (shCTR), the shZEB1.1 or the shZEB1.2.

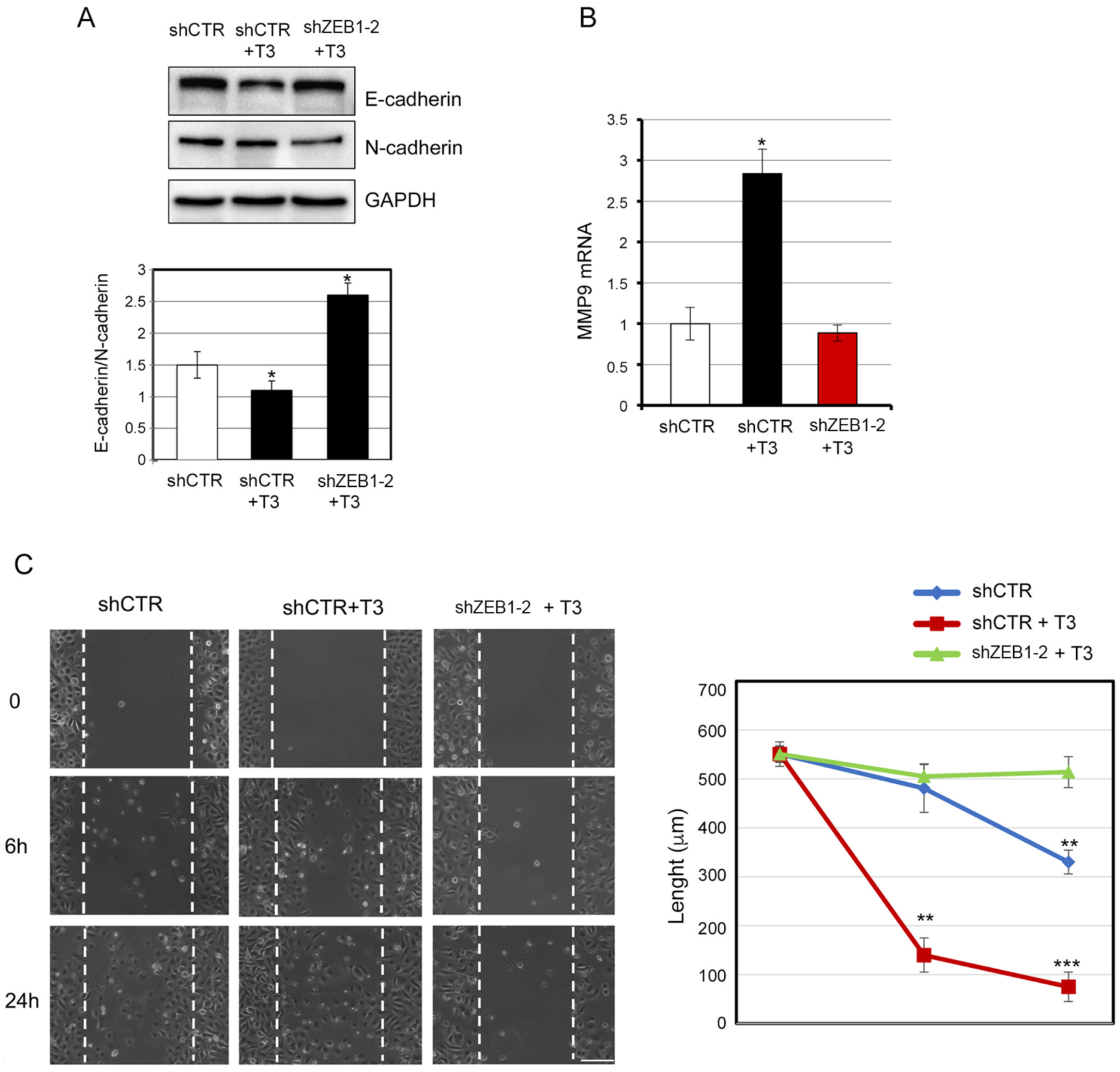

Supplementary Figure 14. *ZEB-1 silencing attenuates T3-dependent EMT.* **(A)** Expression of E-cadherin and N-cadherin in SCC cells treated with T3 in the presence of a scramble (shCTR) or the shZEB1.2. **(B)** mRNA levels of MMP9 was measured in the same cells as in A. **(C)** Wound scratch assay in SCC cells treated or not with 30nM T3 and transfected with shCTR or shZEB1.2.

Supplementary Table 1: List of Oligonucleotides

**Oligonucleotides used for real-time PCR**		
**Gene**	**Forward primer (5' → 3')**	**Reverse primer (5' → 3')**
Cyclophilin A (CypA)	CGCCACTGTCGCTTTTCG	AACTTTGTCTGCAAACAGCTC
CYCLOPHILIN A (CYPA)	AGTCCATCTATGGGGAGAAATTTG	GCCTCCACAATATTCATGCCTTC
Dio2	CTTCCTCCTAGATGCCTACAAAC	GGCATAATTGTTACCTGATTCAGG
DIO2	CTCTATGACTCGGTCATTCTGC	TGTCACCTCCTTCTGTACTGG
Dio3	CCGCTCTCTGCTGCTTCAC	CGGATGCACAAGAAATCTAAAAGC
DIO3	CCTGGGACTCTGCTTCTGTAAC	GGGGTGTAAGAAAATGCTGTAGAG
E-cadherin (Cdh1)	CGTCCTGCCAATCCTGATGA	ACCACTGCCCTCGTAATCGAAC
E-CADHERIN (CDH1)	TGTCGACCGGTGCAATCTT	GGCGCCACCTCGAGAGA
Krt14	GATGTGACCTCCACCAACCG	CCATCGTGCACATCCATGAC
Krt6	TCGTGACCCTGAAGAAGGATGTA	CCTTGGCTTGCAGTTCAACTT
Krt8	ACAACAAGTTCGCCTCCTTC	TCTCCATCTCTGTACGCTTGT
MMP2	AAGGATGGCAAGTACGGCTT	TCATCGTAGTTGGCTGTGGT
MMP3	CTCCAACCGTGAGGAAAATC	CATGGAATTTCTCTTCTCATCAAA
MMP7	GGGGACTCCTACCCATTTGA	TTAGGATCAGAGGAATGTCC
MMP9	CCTGGAGACCTGAGAACCAATC	CGGCAAGTCTTCCGAGTAGT
MMP13	TCTTGTTGCTGCGCATGAGT	AAGGGTCACATTTGTCTGGC
N-cadherin (Cdh2)	ACAGTGGAGCTCTACAAAGG	CTGAGATGGGGTTGATAATG
N-CADHERIN (CDH2)	ACAGTGGCCACCTACAAAGG	CCGAGATGGGGTTGATAATG
TWIST	CCAGCTCCAGAGTCTCTAGA	GCCAGGTACATCGACTTCCT
VIM	GAACCTGCAGGAGGCAGAAG	CATCTTAACATTGAGCAGGTC
**Oligonucleotides used for ChIP analysis**		
**Gene**	**Forward primer (5' → 3')**	**Reverse primer (5' → 3')**
VIM	TGGTTCAGTCCCAGGCGGAC	CATGGTCCCGTTACTTCAGC
ZEB1	CGAGCATTTAGACACAAGCGA	CACTCACCGTTATTGCGCC
**Oligonucleotides used for genome analysis**		
**Gene**	**Forward primer (5' → 3')**	**Reverse primer (5' → 3')**
DIO3	GAGTCTCCCGCCAATTGAAG	AGCCCACCAAGTTCAGTCAA
Dio3	GTCTGGCTAACTTGAGACTCTGCT	TTGTCTTAGAACTAATCCCTTC
Dio2	TCAGAAGGAGACATTCTATTTC	AGGACAGAATCACTTCTTTCGCAA

Supplementary Table 2: List of Antibodies

ANTIBODIES	SOURCE	IDENTIFIER	DILUITION
Mouse monoclonal anti- E-cadherin	BD Biosciences	610181	1:500 IF<br>1:1000 WB
Rabbit polyclonal anti- N-cadherin	Elabscience	E-AB-32170	1:500 WB
Rabbit monoclonal anti-Vimentin	ABCAM	ab-92547	1:2000 WB<br>1:1000 IF
Rabbit polyclonal anti-αTubulin	Santa Cruz Biotecnology	SC-5546	1:10000 WB
Mouse monoclonal anti-αTubulin	Santa Cruz Biotecnology	SC-8035	1:10000 WB
Mouse monoclonal anti-FlagM2	SIGMA	Cat#F3165	1:1000 IF<br>1:1000 WB
Rabbit polyclonal anti-cytokeratin 14	COVANCE	D14IF01918	1:2000 IF
Rabbit polyclonal anti-CXCR4	ABCAM	ab-2074	1:300 IF
Rabbit polyclonal anti-cytokeratin 6	COVANCE	PRB-169P	1:1000 IF
Rat anti-cytokeratin 8 (TROMA 1)	Hybridoma bank	AB_531826	1:300 IF
Anti-D3 718	Homemade	Homemade	1:500 IF<br>1:500 IHC
Anti-D3 717	Homemade	Homemade	1:500 WB
Anti-Phalloidin-TRITC labeled	SIGMA	77418-1EA	1:2000 IF
Rabbit polyclonal Anti-ZEB1	ABCAM	ab-155249	1:500 WB
Rabbit polyclonal Anti-ZEB1	Novus Bio	NBP1-05987	1:250 IF<br>1:250 IHC
Anti-Thyroid hormone receptor antibody (C3)-Chip Grade	ABCAM	ab-2743	2,5 μg
Anti-Thyroid hormone receptor beta antibody Chip Grade	ABCAM	ab-5622	2,5 μg
Rabbit polyclonal anti-GAPDH	Elabscience	E-AB-20059	1:5000 WB
