# Supplementary material for: Thyroid hormone induces progression and invasiveness of squamous cell carcinomas by promoting a ZEB-1/E-cadherin switch
Source: Nat Commun. 2019 Nov 27;10:5410. doi: 10.1038/s41467-019-13140-2 (PMC6881453; doi:10.1038/s41467-019-13140-2)
Supplement: Supplementary file 3 — Description of Additional Supplementary Files [file 41467_2019_13140_MOESM3_ESM.docx]

**Description of Supplementary Files**

**File Name:** Supplementary Data 1

**Description:** Gene list of the differentially regulated genes in the EMT RT2 profiler TM PCR array.

**File Name:** Supplementary Data 2

**Description:** Clinicopathological characteristics of the study population.
