# Supplementary material for: Thyroid hormone induces progression and invasiveness of squamous cell carcinomas by promoting a ZEB-1/E-cadherin switch
Source: Nat Commun. 2019 Nov 27;10:5410. doi: 10.1038/s41467-019-13140-2 (PMC6881453; doi:10.1038/s41467-019-13140-2)
Supplement: Supplementary file 6 — Reporting Summary [file 41467_2019_13140_MOESM6_ESM.pdf]

nature research

Corresponding author(s): DBPR: NCOMMS-17-26952C

Last updated by author(s): Sep 13, 2019

Reporting Summary

Nature Research wishes to improve the reproducibility of the work that we publish. This form provides structure for consistency and transparency in reporting. For further information on Nature Research policies, see Authors & Referees and the Editorial Policy Checklist .

Statistics

For all statistical analyses, confirm that the following items are present in the figure legend, table legend, main text, or Methods section.

n/a	Confirmed	
☐	☒	The exact sample size (*n*) for each experimental group/condition, given as a discrete number and unit of measurement
☒	☐	A statement on whether measurements were taken from distinct samples or whether the same sample was measured repeatedly
☐	☒	The statistical test(s) used AND whether they are one- or two-sided<br>*Only common tests should be described solely by name; describe more complex techniques in the Methods section.*
☒	☐	A description of all covariates tested
☒	☐	A description of any assumptions or corrections, such as tests of normality and adjustment for multiple comparisons
☐	☒	A full description of the statistical parameters including central tendency (e.g. means) or other basic estimates (e.g. regression coefficient) AND variation (e.g. standard deviation) or associated estimates of uncertainty (e.g. confidence intervals)
☒	☐	For null hypothesis testing, the test statistic (e.g. *F*, *t*, *r*) with confidence intervals, effect sizes, degrees of freedom and *P* value noted<br>*Give P values as exact values whenever suitable.*
☒	☐	For Bayesian analysis, information on the choice of priors and Markov chain Monte Carlo settings
☒	☐	For hierarchical and complex designs, identification of the appropriate level for tests and full reporting of outcomes
☒	☐	Estimates of effect sizes (e.g. Cohen's *d*, Pearson's *r*), indicating how they were calculated

Our web collection on statistics for biologists contains articles on many of the points above.

Software and code

Policy information about availability of computer code

Data collection	Genome Browser, TFBind, X-Tile 3.6.1, Gene Cluster 3.0, Java Tree View 1.1.6r4
Data analysis	Graph Pad Prism V7 Software, Cell F Software Olympus, Imagej 1.45s Software, rt2 profiler pcr array data analysis (QIAGEN).

For manuscripts utilizing custom algorithms or software that are central to the research but not yet described in published literature, software must be made available to editors/reviewers. We strongly encourage code deposition in a community repository (e.g. GitHub). See the Nature Research guidelines for submitting code & software for further information.

Data

Policy information about availability of data

All manuscripts must include a data availability statement. This statement should provide the following information, where applicable:

- Accession codes, unique identifiers, or web links for publicly available datasets
- A list of figures that have associated raw data
- A description of any restrictions on data availability

Provide your data availability statement here.

Field-specific reporting

Please select the one below that is the best fit for your research. If you are not sure, read the appropriate sections before making your selection.

☒ Life sciences ☐ Behavioural & social sciences ☐ Ecological, evolutionary & environmental sciences

For a reference copy of the document with all sections, see nature.com/documents/nr-reporting-summary-flat.pdf

Life sciences study design

All studies must disclose on these points even when the disclosure is negative.

Sample size	Twelve to fifteen mice per group were used in each experiment. The precise number of animals used is given in the figures.
Data exclusions	No data were excluded from the analyses.
Replication	All experiments were performed in triplicate. All attempts at replication were successful.
Randomization	N/A. We did not use randomization to assign animals to experimental groups.
Blinding	Investigators were not blinded to group allocation during experiments.

Reporting for specific materials, systems and methods

We require information from authors about some types of materials, experimental systems and methods used in many studies. Here, indicate whether each material, system or method listed is relevant to your study. If you are not sure if a list item applies to your research, read the appropriate section before selecting a response.

Materials & experimental systems

n/a	Involved in the study	
☐	☒	Antibodies
☐	☒	Eukaryotic cell lines
☒	☐	Palaeontology
☐	☒	Animals and other organisms
☒	☐	Human research participants
☒	☐	Clinical data

Methods

n/a	Involved in the study	
☒	☐	ChIP-seq
☐	☒	Flow cytometry
☒	☐	MRI-based neuroimaging

Antibodies

Antibodies used	All the antibodies used in the study were described in Supplementary Table 4.
Validation	All the antibodies were validated as indicated on the manufacturer's website.

Eukaryotic cell lines

Policy information about cell lines

Cell line source(s)	*State the source of each cell line used.*
Authentication	Cellosaurus SCC-13 (RRID:CVCL_4029); Cellosaurus SCC-011 (RRID:CVCL_5986); Cellosaurus SCC-022 (RRID:CVCL_5991) as indicated in Methods.
Mycoplasma contamination	All cell lines were mycoplasma free
Commonly misidentified lines (See ICLAC register)	No misidentified cell lines were used in the study.

Animals and other organisms

Policy information about studies involving animals; ARRIVE guidelines recommended for reporting animal research

Laboratory animals	sD3KO (K14CreERT-D3fl/fl); sD2KO (K14CreERT-D2fl/fl) and D2-3xFlag mice were C57/B16 strain. Both males and females of 5 weeks were used. All the experiment were performed in accordance with institutional guidelines.
Wild animals	The study did not involve wild animals.
Field-collected samples	The study did not involve samples collected from the field.
Ethics oversight	All animal studies were conducted in accordance with the guidelines of the Ministero della Salute and were approved by the Institutional Animal Care and Use Committee (IACUC, n. 167/2015-PR and n. 354/2019-PR).

Note that full information on the approval of the study protocol must also be provided in the manuscript.

Flow Cytometry

Plots

Confirm that:

- [x] The axis labels state the marker and fluorochrome used (e.g. CD4-FITC).
- [x] The axis scales are clearly visible. Include numbers along axes only for bottom left plot of group (a 'group' is an analysis of identical markers).
- [x] All plots are contour plots with outliers or pseudocolor plots.
- [ ] A numerical value for number of cells or percentage (with statistics) is provided.

Methodology

Sample preparation	Tumors were digested in collagenase I for 2 h at 37°C. Immunostaining was performed using APC-anti-mouse CD34, PE-rat anti human a6-integrin, FITC anti-CD3/GR-1/CD11B/CD45-B220/TER 119, vioBlu-anti mouse CD326 by incubation for 1 hour, as indicated in Methods.
Instrument	FACS Canto2, Becton Dickinson
Software	FACS Canto2 software
Cell population abundance	Sort-purification was carried out using acustom configuration FACS Canto2 cell sorter. ≥2X10*5 of TIC were routinely achieved.
Gating strategy	Living tumour cells were selected by forward scatter, side scatter and doublet discrimination by Hoechst dye exclusion.

- [ ] Tick this box to confirm that a figure exemplifying the gating strategy is provided in the Supplementary Information.
